# Utilization of an Umbilical Hernia as a Camera Port in Robot-Assisted Total Hysterectomy: A Case Report

**DOI:** 10.7759/cureus.102288

**Published:** 2026-01-25

**Authors:** Shohei Wakisaka, Hiroharu Kobayashi, Aimi Oda, Yumi Shimizu, Masahiro Kanamori

**Affiliations:** 1 Gynecology, Seirei Hamamatsu General Hospital, Hamamatsu, JPN; 2 Obstetrics and Gynecology, Seirei Hamamatsu General Hospital, Hamamatsu, JPN

**Keywords:** da vinci system, mesh-free repair, robot-assisted hysterectomy, trocar placement, umbilical hernia

## Abstract

We report a case of robot-assisted total hysterectomy in which an umbilical hernia was utilized as the camera port. A 43-year-old obese woman with atypical endometrial hyperplasia and an incidental umbilical hernia underwent surgery. After resection of omental contents from the hernia sac, a balloon trocar was placed in the umbilicus and used as a camera port. The hysterectomy was safely completed, and hernia repair followed. To our knowledge, only limited reports have described this approach in robotic surgery.

This case highlights the technical feasibility of using an umbilical hernia for robotic port placement, though careful postoperative monitoring is warranted due to the risk of recurrence without mesh repair.

## Introduction

Umbilical hernia is frequently encountered in obese patients and may complicate port placement in minimally invasive gynecologic surgery. In standard robotic-assisted gynecologic surgery, a camera port is created at or above the umbilicus. In patients with umbilical hernias, a camera port at the umbilicus cannot be created as is. Reports of minimally invasive surgery in patients with umbilical hernias exist outside gynecology, though they are few in number. Laparoscopic cholecystectomy with simultaneous hernia repair has been reported, but few details were provided [1]. In robotic surgery, Kim et al. described reducing an umbilical hernia and using it as a port during prostatectomy [2].

We present a case of robot-assisted total hysterectomy, where the umbilical hernia was used as the camera port, demonstrating a novel and practical approach in selected patients.

## Case presentation

The patient was a 43-year-old woman, gravida 0, with a height of 160 cm and a weight of 83 kg (BMI, 32 kg/m²). She consulted a previous doctor due to irregular menstruation and was referred to our hospital because of abnormal endometrial cytology. Transvaginal ultrasound showed that the uterus was the size of an egg, the endometrium was 12 mm thick, there was a Naboth cyst, and there were no abnormalities in the bilateral adnexa.

**Figure 1:**
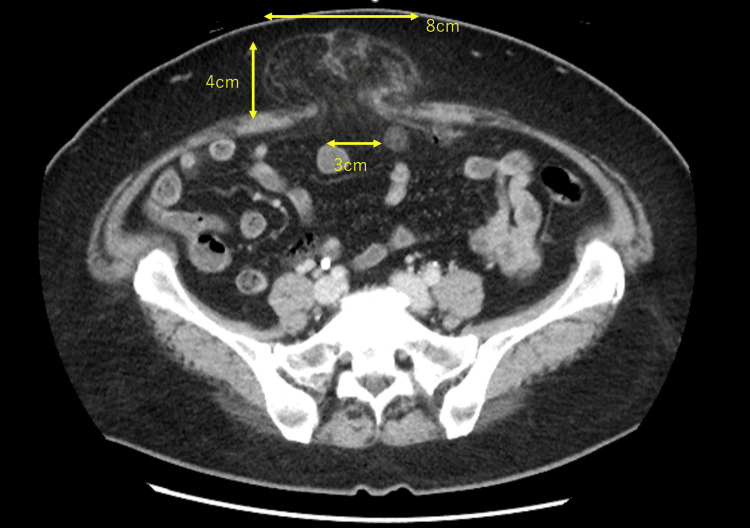
Computed tomography confirmed an umbilical hernia, with the hernia contents suspected to be the omentum.

The endometrial suction biopsy showed atypical endometrial hyperplasia. Considering the possibility of cancer, we performed imaging studies. Chest and abdominal contrast computed tomography showed no distant metastasis or enlarged lymph nodes, but a hernia was found in the umbilical region (3 cm at the hernia orifice, 4 cm in the depth of the hernia sac, and 8 cm in its spread; Figure 1). The contents of the hernia sac were thought to be omentum. The patient’s umbilical region did not bulge, but she felt discomfort when touching the area below the navel. Contrast magnetic resonance imaging showed no soft tissue mass in the uterine cavity.

For patients without fertility preservation desires, hysterectomy is the first-line treatment for atypical endometrial hyperplasia. Endometrial cancer is found in 30%-50% of hysterectomized uteri removed due to an atypical endometrial hyperplasia diagnosis [3]. For early-stage endometrial cancer, the five-year survival rates for open surgery and laparoscopic surgery are nearly equivalent [4]. Furthermore, for the surgical treatment of endometrial cancer, long-term prognosis is comparable between robot-assisted surgery and laparoscopic surgery [5].

**Figure 2:**
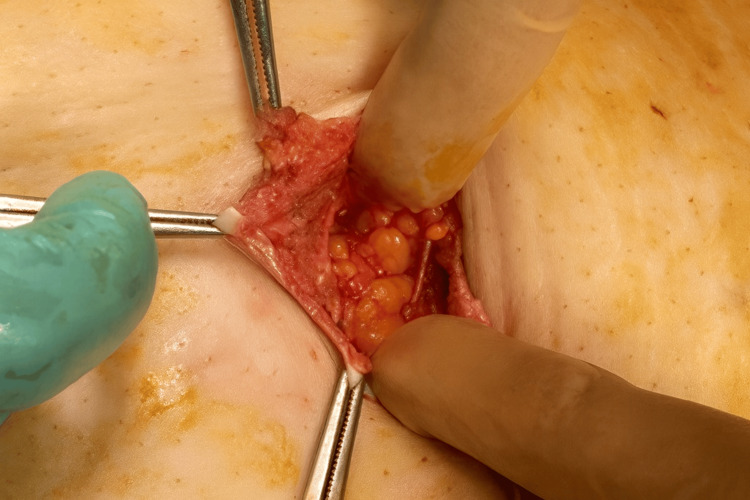
Incision at the umbilicus revealed the omentum within the hernia sac.

**Figure 3:**
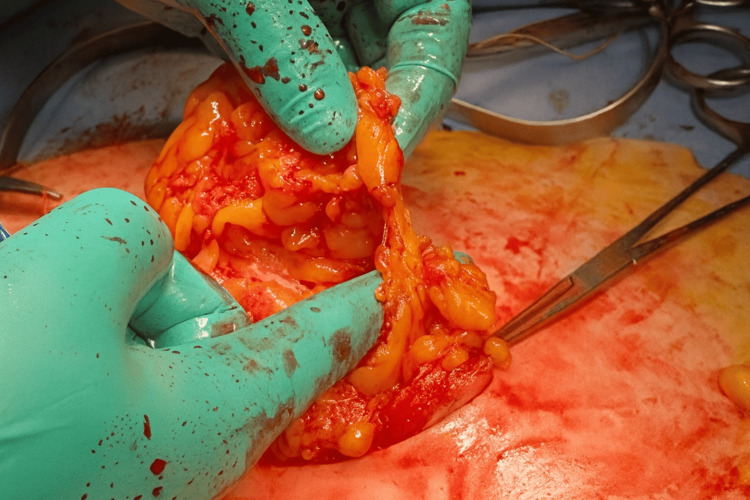
The omentum within the hernia sac was exteriorized and resected.

**Figure 4:**
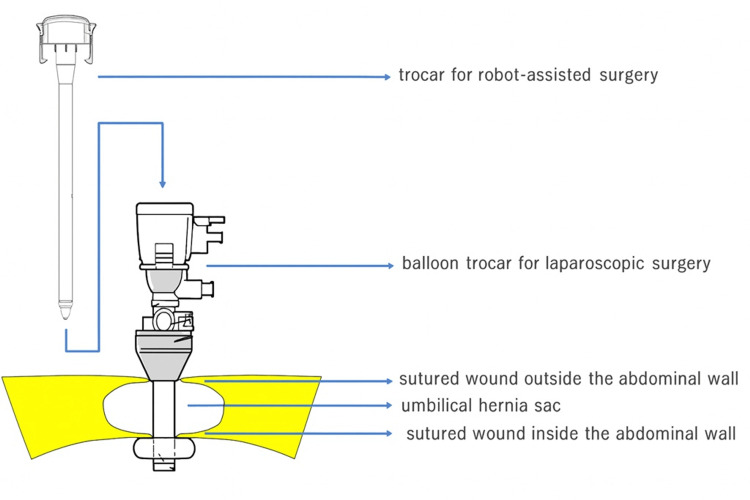
The hernia sac was left in situ and used as a trocar insertion site for the da Vinci system. This figure was created by the authors and is an original image.

**Figure 5:**
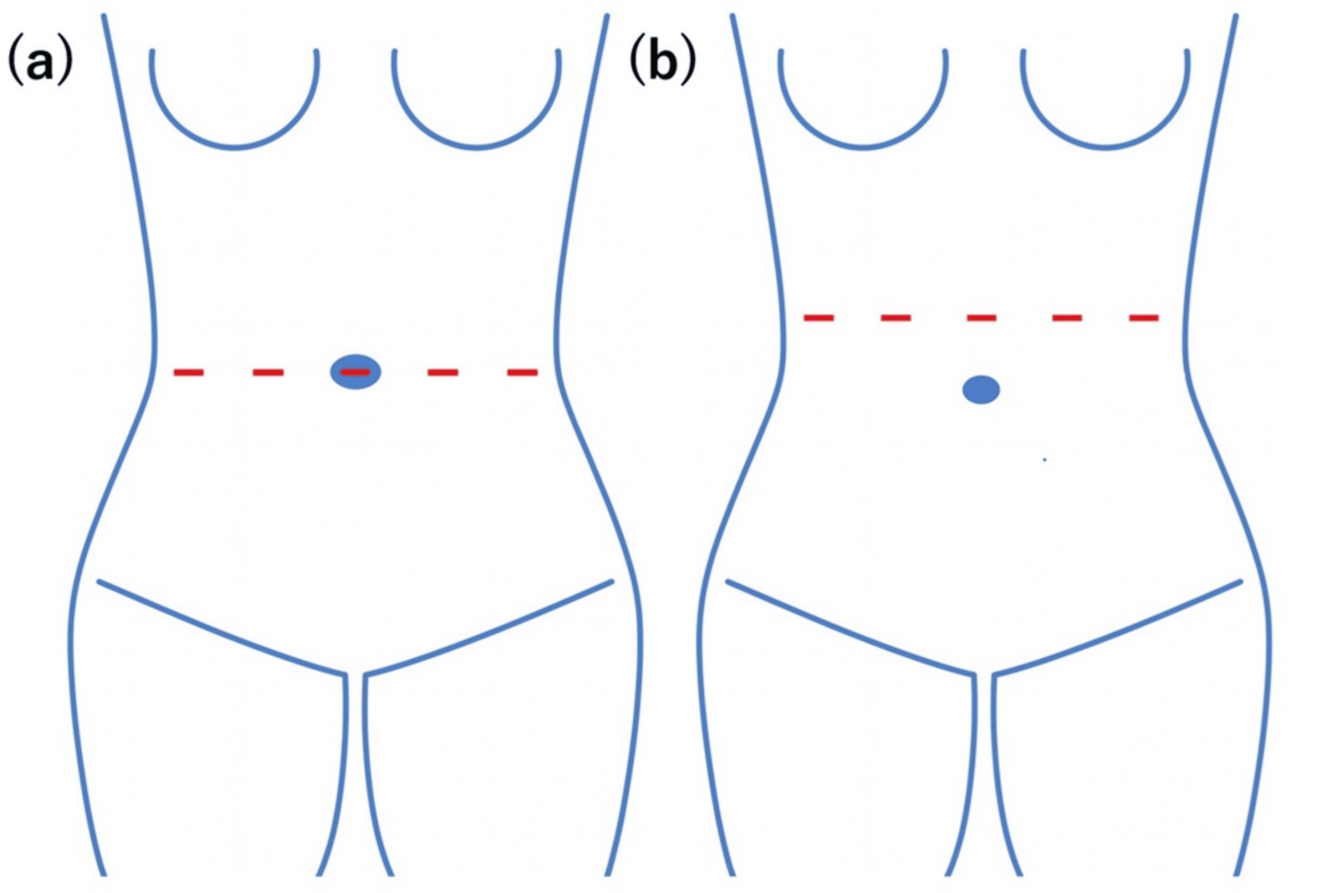
(a) The standard da Vinci port configuration routinely used at our institution. (b) The da Vinci port configuration used at our institution for cases with a large uterus or a short umbilicus-to-pubis distance. These figures were created by the authors and are original images.

We selected robot-assisted hysterectomy as the treatment method for this patient. A camera port for robot-assisted surgery was created using the umbilical hernia containing the omentum as follows: the navel was incised vertically for about 4 cm, and the omentum that had escaped into the hernia sac was identified (Figure 2). This herniated omentum was excised and removed (Figure 3), and the hernia orifice, with a diameter of about 4 cm, was identified. The incised skin and hernia orifice were sutured to reduce their size, and a 12 mm diameter balloon trocar sleeve (Kii Balloon Blunt Tip System, 12 x 100 mm, Aesculap, Tuttlingen, Germany) was attached here. A trocar for robot-assisted surgery (da Vinci system, Intuitive, Sunnyvale, CA, USA) was inserted inside this trocar (Figure 4) and used as a camera port, with three additional ports for the da Vinci system created at intervals of 8 cm on both sides (Figure 5a). Pneumoperitoneum of 10 mmHg was maintained using the Air Seal system (ConMed, Largo, FL, USA).

Robot-assisted hysterectomy and bilateral salpingectomy were performed. The removed specimen was retrieved through the vagina, and the vaginal stump was sutured with absorbable thread. Intraoperative frozen section diagnosed atypical endometrial hyperplasia, so both ovaries were preserved. After the trocar was removed, the hernia orifice (umbilical ring) was exposed circumferentially, and the hernia sac, which was about 6 cm in diameter, was resected. The hernia orifice was sutured with non-absorbable thread (No. 1 Ethibond, Ethicon, Somerville, NJ, USA) in a continuous suture, and the navel was reconstructed with absorbable thread (4-0 PDS, Ethicon).

The surgery lasted 3 hours and 58 minutes, with a blood loss of 6 mL. The hospital stay was five days. There were no intraoperative or postoperative complications. Pathological examination revealed endometrial cancer, specifically endometrioid carcinoma, grade 1, stage IA according to the FIGO staging system, with no myometrial invasion, lymphovascular space invasion, or cervical stromal invasion [6]. Currently, she continues outpatient follow-up without additional treatment.

## Discussion

In robot-assisted total hysterectomy in our hospital, a camera trocar is usually attached to the navel, and three ports for the da Vinci system are created 7 to 8 cm apart on either side (Figure 5a). In cases where the patient is small, the distance from the navel to the pubis is short, or the uterus is large, trocar ports are created a few centimeters above the navel (Figure 5b).

In this case, the patient was obese, but she was not small in stature (160 cm), and her uterus was not large, so if it were not for the umbilical hernia, the camera port would have been created at the navel. However, due to the presence of an umbilical hernia, creating a port at a position distant from the umbilicus was also considered.

It was suspected that the omentum was involved in the umbilical hernia, and leaving the hernia unrepaired would obstruct the endoscopic view, even if a port were created at a different location from the umbilicus. Therefore, we devised a method to create a camera port at the umbilicus while excluding the omentum from the umbilical hernia and leaving the hernia sac intact (Figure 4).

After total hysterectomy, the hernia sac was resected, and the hernia orifice was sutured with non-absorbable sutures. The 2020 EHS/AHS guideline recommends mesh repair for umbilical and epigastric hernias to reduce recurrence; sutured repair may be considered only for small defects (<1 cm) in shared decision-making. For symptomatic medium-sized defects (>1-4 cm), an open repair with a preperitoneal flat mesh is suggested [7].

In this case, the hernia orifice of the umbilical hernia was approximately 4 cm, making it a case where mesh repair would typically be preferred. While we need cooperation with a surgeon specializing in hernias for umbilical hernia repair, this case was suspected to be malignant, and surgery was scheduled as semi-urgent for oncological priority. Due to time constraints for surgery, a mesh-free approach was initially selected, and we decided to perform surgery using mesh later, if necessary.

Reports on minimally invasive surgery for patients with an umbilical hernia are very limited. Gundogdu et al. conducted a retrospective review of 71 cases in which the umbilical hernia was repaired simultaneously during laparoscopic cholecystectomy [1]. However, they did not report the specific methods used. In robot-assisted surgery, only one report by Kim et al. was found in a PubMed search [2]. They reduced the umbilical hernia and used it as a camera port during robot-assisted prostatectomy. This case demonstrated that it is possible to remove the contents of an umbilical hernia while leaving the hernia sac intact and using it as a da Vinci port. However, since a mesh-free repair method was chosen for the umbilical hernia, the risk of recurrence is high, and careful follow-up will be necessary in the future. No proprietary tools, scales, or scoring systems were used in this study.

## Conclusions

This case demonstrates that an incidental umbilical hernia can be safely and effectively utilized as a camera port during robot-assisted total hysterectomy, after appropriate management of the hernia contents. By resecting the herniated omentum and temporarily reducing the hernia orifice, standard robotic port placement was maintained without compromising surgical safety or visualization. This approach may represent a practical option in selected patients, particularly when the umbilicus is the preferred site for camera port placement. However, when mesh-free hernia repair is performed in cases with a large hernia defect, careful long-term postoperative follow-up is essential due to the risk of recurrence. Further accumulation of cases is needed to establish the safety, reproducibility, and optimal indications for this technique in gynecologic robotic surgery.
